# Mapping factors associated with emotional and behavioral problems among preschool children: A scoping review protocol

**DOI:** 10.1371/journal.pone.0353520

**Published:** 2026-07-15

**Authors:** Esme Anggeriyane, Siti Yuyun Rahayu Fitri, Meita Dhamayanti, Windy Rakhmawati, Bella Mardya

**Affiliations:** 1 Doctoral Study Program in Medical Science, Faculty of Medicine, Universitas Padjadjaran, Bandung, West Java, Indonesia; 2 Department of Pediatric Nursing, Faculty of Nursing and Health Sciences, University of Muhammadiyah Banjarmasin, Banjarmasin, South Borneo, Indonesia; 3 Department of Pediatric Nursing, Faculty of Nursing, Universitas Padjadjaran, Sumedang, West Java, Indonesia; 4 Department of Child Health, Faculty of Medicine, Universitas Padjadjaran, Bandung, West Java, Indonesia; 5 Department of Pediatric Nursing, Faculty of Medicine, Public Health and Nursing, University of Gadjah Mada, Yogyakarta, Central Java, Indonesia; University of Porto Faculty of Psychology and Educational Sciences: Universidade do Porto Faculdade de Psicologia e de Ciencias da Educacao, PORTUGAL

## Abstract

Emotional and behavioral problems in preschool children are increasingly regarded as a major public health concern, particularly as early life challenges can lead to long-lasting difficulties in later childhood and adulthood. Although numerous studies have examined the factors associated with EBPs, the evidence remains fragmented across disciplines, study designs, and measurement approaches, limiting a coherent understanding. This scoping review aims to systematically map and synthesize factors associated with emotional and behavioral problems in preschool children. This scoping review will follow the methodological framework proposed by Peters (2020), in accordance with the Joanna Briggs Institute (JBI) guidance, and documented in accordance with the Preferred Reporting Items for Systematic Reviews and Meta-Analyses for Scoping Reviews (PRISMA-ScR) guidelines. A systematic search will be undertaken in PubMed, Scopus, Web of Science (WoS), the Cochrane Library, Embase, and EBSCOhost (via MEDLINE and CINAHL) for studies published from 2000 onwards. Qualitative, quantitative, and mixed-methods studies, with no language restrictions, will be included. Study selection and data charting will be independently conducted by at least two reviewers, with disagreements resolved through discussion. Data will be synthesized using a descriptive analytical approach, with factors categorized into genetic, biological, psychological, and environmental domains. Findings will be presented through narrative synthesis and summary tables, with frequency mapping and thematic grouping used to identify patterns, variations in measurement approaches, and evidence gaps. This review will provide a comprehensive overview of how factors associated with EBPs in preschool children have been studied and will inform future research, policy, and early prevention strategies.

## Introduction

Mental health problems in children represent a major global public health concern. The prevalence estimates range from 10 to 20% [1]. Among these, emotional and behavioral problems (EBPs) are among the most reported conditions and can emerge as early as the preschool period [2–5]. Early childhood is widely recognized as a critical developmental window, characterized by rapid growth across cognitive, emotional, linguistic, and social domains, which increases children’s vulnerability to emotional and behavioral regulation disruptions [6,7].

EBPs in preschool children refer to persistent difficulties in age-appropriate and adaptive regulation of emotions and behaviors, encompassing both internalizing and externalizing domains. Internalizing problems include symptoms such as anxiety, depression, emotional withdrawal, and excessive fear, whereas externalizing problems include hyperactivity, inattention, aggression, oppositional behavior, and difficulties in social interaction, such as sharing and cooperating with peers [8–17]. These problems frequently co-occur and may interact with other developmental conditions, complicating early identification and intervention efforts [18–20].

Population-based studies indicate that EBPs are relatively common among preschool children, with prevalence estimates ranging from 7% to 25%, depending on the assessment tools, informants, and settings [21]. Evidence across countries further highlights the widespread and cross-cultural nature of these problems [21–23]. More detailed epidemiological findings from studies across countries and specific contexts further illustrate this variability. For example, evidence from Malaysia has reported a prevalence of around 8.4% in preschool populations, with domain-specific patterns such as peer problems (19.7%), hyperactivity (5.6%), and emotional problems (6.8%) varying by context and measurement approach [23]. EBPs may persist and develop into more severe emotional and behavioral disorders over time if not identified and addressed early [21,22]. This variability in prevalence and measurement underscores the need for a systematic and integrative synthesis of evidence to better understand the complexity of EBPs in preschool children.

The consequences of EBPs extend beyond early childhood and can affect multiple developmental domains. EBPs manifest as internalizing and externalizing symptoms that interfere with daily functions in the short term [24,25]. Longitudinal evidence suggests that early EBPs are associated with adverse outcomes in later life, including poor academic achievement, impaired social relationships, reduced quality of life, and lower productivity in adulthood [24,26–31]. These findings underscore the importance of early identification and comprehensive understanding of factors associated with EBPs during the preschool period.

A growing body of research has examined factors associated with EBPs across different populations and settings. A wide range of contributing factors have been identified, including family dynamics, parenting practices, socioeconomic conditions, child temperament, and broader environmental influences [6,21,24,32–34]. Biological and developmental factors, such as prenatal exposure, neurological conditions, and genetic predispositions, have also been implicated [21,26,30,35,36]. However, much of the existing literature focuses on isolated factors, specific domains, or older age groups, thereby limiting a comprehensive understanding of EBPs within the unique developmental context of preschool children.

Previous review studies have examined EBPs within specific contexts or populations, such as disordered eating behavior, foster care settings, or parental stress, often focusing on limited sets of factors or age groups. Most of these studies have been conducted as systematic reviews or meta-analyses with a relatively narrow scope, rather than as comprehensive scoping reviews designed to map the full breadth of evidence across multiple domains [37–41]. However, these reviews are limited in their ability to provide an integrative synthesis because of their narrow scope, focus on specific populations or conditions, and reliance on methodologies that prioritize effect estimation over comprehensive evidence mapping. While these studies provide valuable insights, they do not offer a unified mapping of factors across domains. Inconsistencies in the categorization of influencing factors and variability in methodological approaches have contributed to fragmentation in the existing evidence base. To the best of our knowledge, no prior scoping review has systematically mapped genetic, biological, psychological, and environmental factors within a unified analytical framework specifically for preschool children.

To address this gap, the present study adopts a theoretically informed framework grounded in the biopsychosocial model, which conceptualizes health and behavior as the result of dynamic interactions among biological, psychological, and social determinants [42–45]. The inclusion of genetic, biological, psychological, and environmental domains reflects an effort to operationalize the biopsychosocial framework into analytically distinct yet interrelated categories for evidence mapping. The biological domain is further differentiated within this framework to distinguish genetic factors from broader biological influences, enhancing conceptual precision and analytical clarity. This approach is further supported by a developmental perspective, including Bronfenbrenner’s bioecological model, which emphasizes the interaction between individual characteristics and environmental contexts across time [46–48].

A preliminary search of the Cochrane Database of Systematic Reviews, Open Science Framework, and JBI Evidence Synthesis was conducted in September 2025, which identified no ongoing or completed scoping reviews that comprehensively map factors associated with EBPs in preschool children. Notably, existing reviews have not systematically mapped the breadth of factors across multiple domains within the preschool developmental stage, nor have they employed a scoping review approach to capture the heterogeneity of evidence sources, study designs, and conceptualizations. Therefore, this scoping review aims to map and synthesize the existing literature on factors associated with EBPs in preschool children across genetic, biological, psychological, and environmental domains. This further seeks to describe how these factors have been examined and reported across studies, without implying causal relationships. This review provides an integrated and theoretically grounded overview of these factors to enhance conceptual clarity, identify research gaps, and inform future research, policy, and early intervention strategies. Ultimately, this work is expected to support the development of more targeted, evidence-informed approaches for the early identification and prevention of EBPs in preschool populations, both in clinical practice and at the public health level.

### Objective

This scoping review aims to map and synthesize the existing literature on factors associated with emotional and behavioral problems in preschool children. This review seeks to describe how these factors have been examined and reported across studies, without implying causal relationships.

### Review questions

What factors have been associated with emotional and behavioral problems in preschool children?

Research sub-questions are:

1. What genetic factors have been reported to be associated with emotional and behavioral problems in preschool children?
2. What biological factors have been reported to be associated with emotional and behavioral problems in preschool children?
3. What psychological factors have been reported to be associated with emotional and behavioral problems in preschool children?
4. What environmental factors have been reported to be associated with emotional and behavioral problems in preschool children?

## Materials and methods

This scoping review will be followed by the Joanna Briggs Institute (JBI) scoping review methodology and will adhere to the Preferred Reporting Items for Systematic Review and Meta-Analyses Extension for Scoping Reviews (PRISMA-ScR) guidelines to ensure methodological rigor and transparent reporting [49,50] (S1 File). The review process will be conducted in sequential stages, including systematic searching, study selection, data extraction, and data synthesis and presentation.

### Protocol and registration

This protocol was registered in the Open Science Framework (OSF) at https://osf.io/35rn9 on October 6, 2025.

### Eligibility criteria

The inclusion criteria were formulated according to the Population, Concept, Context (PCC) framework and the types of evidence sources.

#### Population.

This review will include studies involving preschool children aged 3–6 years old [51]. Studies will be eligible regardless of baseline status, provided that emotional and/or behavioral problems are examined as outcomes or phenomena of interest using standardized screening or assessment tools such as the Child Behavior Checklist (CBCL), Strengths and Difficulties Questionnaire (SDQ), Pediatric Symptom Checklist-17 (PSC-17), or comparable instruments. Studies that do not clearly distinguish preschool-aged children or that focus exclusively on younger or older age groups will be excluded.

#### Concept.

The concept refers to factors associated with EBPs, conceptualized across genetic, biological, psychological, and environmental domains without implying causal relationships [52]. Emotional and behavioral problems are broadly defined to capture subclinical difficulties rather than formally diagnosed psychiatric disorders. Therefore, studies focusing exclusively on disorders diagnosed according to the International Statistical Classification of Diseases and Related Health Problems (ICD) and the Diagnostic and Statistical Manual of Mental Disorders (DSM) will be excluded.

#### Context.

This review will include studies conducted in any geographical setting and across a range of environments, including home, early childhood education settings, community and social environments, and public or community health services, in which preschool children live and develop.

#### Types of sources.

This review will include original research articles that employ qualitative, quantitative, or mixed-methods designs. Grey literature, including editorials, commentaries, conference abstracts, theses, dissertations, blogs, and opinion papers, will be excluded because these sources are not consistently subject to rigorous peer-review processes and may vary in methodological transparency and quality. Studies published in any language will be considered to minimize language bias and ensure comprehensive coverage of relevant evidence. The handling of non-English studies will be integrated within the data extraction procedures. No language restriction will be applied. For studies published in languages other than English, the initial translation will be conducted using a reliable translation tool (e.g., Google Translate), followed by verification by a reviewer with proficiency in the respective language, where possible. Key sections (e.g., methods and results) will be cross-checked when necessary to ensure data extraction accuracy and consistency. To reflect the contemporary conceptualization of EBPs, the publication period will be limited to studies published from 2000 onwards.

### Search strategy

Two reviewers will design the search strategy to identify studies relevant to the Population, Concept, and Context. A comprehensive database search will be undertaken across the following seven databases: PubMed, Scopus, Web of Science (WoS), the Cochrane Library, Embase, and EBSCOhost (via MEDLINE and CINAHL). As part of the preparatory phase, a preliminary search was conducted in PubMed to identify relevant keywords and index terms related to preschool children, emotional and behavioral problems, and associated factors (S2 File). These terms informed the development of the final search strategy, which will incorporate controlled vocabulary (e.g., MeSH terms) and free-text terms across databases, including “title-abstract-keyword”, “topic”, and “text word fields” as applicable. The search strategy was peer-reviewed by subject experts to enhance methodological validity. No language restrictions will be applied, and studies published from 2000 onwards will be included.

### Study selection

All retrieved records will be imported into EndNote v.21 (Clarivate Analytics, PA, USA), and duplicates will be removed following the search process [53]. Study selection will be conducted in two stages: (1) title and abstract screening and (2) full-text screening. Two reviewers (WR and BM) will independently screen the records following a pilot calibration exercise to ensure consistency with the predefined inclusion criteria. Before the screening process, a calibration exercise will be conducted using a random sample of approximately 5–10% of the retrieved records, consistent with the methodological guidance of the scoping review. Both reviewers will independently screen these records, followed by a discussion to resolve discrepancies and refine the inclusion and exclusion criteria. If a high level of agreement is not achieved (e.g., below 90%), further calibration will be performed before proceeding [53]. This process serves as training and ensures a shared understanding of screening procedures.

Inter-rater agreement will be assessed using percentage agreement (target ≥90%) as the primary indicator of consistency during calibration [53]. Cohen’s kappa coefficient will also be calculated as a complementary measure to account for agreement beyond chance. Interpretation of the kappa value will follow Landis and Koch, where values of 0.61–0.80 indicate substantial agreement and values above 0.80 indicate almost perfect agreement [54]. Any disagreements will be resolved through discussion and, if necessary, consultation with a third review (SYRF), as recommended in the JBI Manual for Evidence Synthesis [50]. The reasons for excluding full-text articles will be documented and reported in the final review. A PRISMA flow diagram will be used to present the study selection process (S1 Fig). As this manuscript reports a study protocol, these stages have not yet started at the time of submission.

### Data extraction

Data extraction will be conducted independently by two reviewers using a standardized data charting form developed in accordance with JBI guidance [50] (S3 File). The data charting form will be pilot-tested on approximately 5–10 studies to ensure clarity and consistency prior to full extraction [53]. Two reviewers will independently extract data from these studies and subsequently meet to compare results, resolve discrepancies, and refine the charting form, as recommended in practical guidance for scoping review protocols [55]. Extracted information will include author and year, study design, participant characteristics, measurement tools, domain classification, and key findings, and will be managed in Microsoft Excel. An additional calibration step will be conducted during the pilot data extraction phase to ensure consistency across reviewers in data charting and interpretation. Any discrepancies between reviewers will be resolved through discussion and consensus, and if necessary, consultation with a third reviewer [50]. Authors of included studies may be contacted for clarification where required. Any modifications to the data extraction form will be documented and reported.

### Data analysis and presentation

Data will be descriptively summarized and mapped across four domains: genetic, biological, psychological, and environmental factors. The findings will be presented using narrative synthesis and summary tables in accordance with the JBI scoping review methodology. Given the inclusion of both quantitative and qualitative studies, a descriptive analytical approach will be employed, whereby factors associated with EBPs are categorized across the predefined domains. To enhance analytical rigor, frequency mapping will be used to quantify the distribution of evidence across domains, while thematic grouping will be applied to categorize the associated factors. A thematic synthesis approach will be used for qualitative studies, in which data will be inductively coded to identify recurring concepts, grouped into themes, and subsequently mapped onto the four conceptual domains. An evidence gap map will be developed to identify areas with limited or absent evidence. No formal critical appraisal of the included studies will be conducted, in accordance with the objectives of a scoping review.

### Study status and projected timeline

This scoping review has not yet commenced at the time of protocol submission in terms of formal study selection and data extraction. A preliminary PubMed search was conducted on September 4, 2025, to refine keywords and index terms. The full database search will commence following publication of this protocol. Title and abstract screening is planned for May to June 2026, followed by full-text screening and data extraction from June to July 2026. Data synthesis and reporting are expected to be completed in Augst 2026.

## Discussion

This scoping review will provide a comprehensive overview of how EBPs in preschool children have been examined in relation to genetic, biological, psychological, and environmental factors. The proposed framework for mapping the existing literature will clarify how these domains are conceptualized and operationalized across studies, using rigorous and transparent methods and documentation to avoid duplication and support updates [56]. The alignment between findings and review questions supports overall review coherence [57]. This scoping review is informed by the existing evidence base to support the development of a more solid evidence-based approach [58].

In particular, it is important to map factors associated with EBPs in preschool children. The findings of this scoping review are expected to be relevant to the general public, the scientific community, practitioners, professional associations, and policy-making organizations. The results may contribute to advancing knowledge and supporting nurses and other health professionals in adapting their interpersonal and clinical approaches to meet the needs of preschool children and to support early prevention of EBPs. Given the broad relevance of the topic, the findings will be disseminated in multiple accessible formats, including scientific publications, presentations at academic conferences, and dissemination through relevant digital platforms [59].

Importantly, this review does not aim to establish causal relationships. Instead, it aims to describe the scope, nature, and distribution of existing evidence. Thereby supporting the development of more integrated, developmentally informed research and prevention-oriented approaches.

## Supporting information

S1 Fig PRISMA flow diagram. (JPG)

S1 File Preferred Reporting Items for Systematic reviews and Meta-Analyses extension for Scoping Reviews (PRISMA-ScR) Checklist. (DOCX)

S2 File Search strategy for PubMed. (DOCX)

S3 File Data extraction form. (DOCX)
