# Supplementary figures and images for: Mapping factors associated with emotional and behavioral problems among preschool children: A scoping review protocol
Source: PLoS One. 2026 Jul 15;21(7):e0353520. doi: 10.1371/journal.pone.0353520 (PMC13372158; doi:10.1371/journal.pone.0353520)

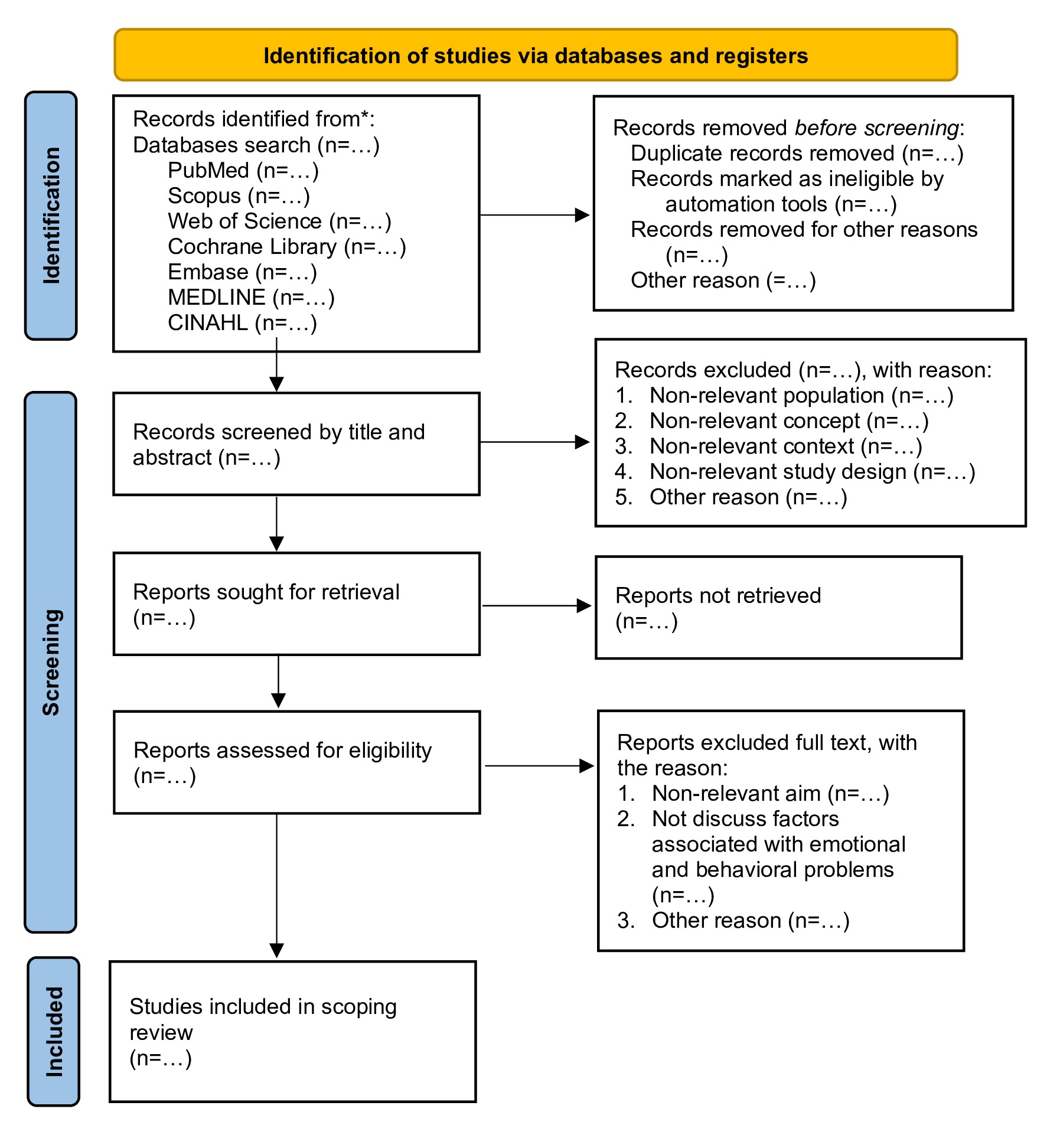

Supplement: S1 Fig — (JPG) [file pone.0353520.s001.jpg]
