# Supplementary material for: Mapping factors associated with emotional and behavioral problems among preschool children: A scoping review protocol
Source: PLoS One. 2026 Jul 15;21(7):e0353520. doi: 10.1371/journal.pone.0353520 (PMC13372158; doi:10.1371/journal.pone.0353520)
Supplement: S2 File — (DOCX) [file pone.0353520.s003.docx]

### S2 File. Sample search strategy for PubMed

Search conducted on September 4, 2025

**PubMed**

| **Search Number** | **Query** | **Results** |
| --- | --- | --- |
| #6 | ((((((child, preschool[MeSH Terms]) OR ("preschool child"[Text Word])) OR ("preschool children"[Text Word])) OR (Preschooler*[Text Word])) OR ("early childhood"[Text Word])) AND ((((((((((risk factors[MeSH Terms]) OR (risk[MeSH Terms])) OR (protective factors[MeSH Terms])) OR (risk assessment[MeSH Terms])) OR (risk*[Text Word])) OR (protective*[Text Word])) OR (predict*[Text Word])) OR (determinant*[Text Word])) OR (factor*[Text Word])) AND (((((((problem behavior[MeSH Terms]) OR ("behavioral problem*"[Text Word])) OR ("emotional problem*"[Text Word])) OR ("problem*, behavioral"[Text Word])) OR ("emotional and behavioral problems"[Text Word])) OR ("behavioural problem*"[Text Word])) OR ("emotional and behavioural problems"[Text Word])))) AND ((((((((community health services[MeSH Terms]) OR (community health nursing[MeSH Terms])) OR ("community health care"[Text Word])) OR ("community health centers"[Text Word])) OR (home[Text Word])) OR (kindergarten[Text Word])) OR (environment[Text Word])) OR (day care[Text Word])) | 615 |
| #5 | (((((((((risk factors[MeSH Terms]) OR (risk[MeSH Terms])) OR (protective factors[MeSH Terms])) OR (risk assessment[MeSH Terms])) OR (risk*[Text Word])) OR (protective*[Text Word])) OR (predict*[Text Word])) OR (determinant*[Text Word])) OR (factor*[Text Word])) AND (((((((problem behavior[MeSH Terms]) OR ("behavioral problem*"[Text Word])) OR ("emotional problem*"[Text Word])) OR ("problem*, behavioral"[Text Word])) OR ("emotional and behavioral problems"[Text Word])) OR ("behavioural problem*"[Text Word])) OR ("emotional and behavioural problems"[Text Word])) | 12,192 |
| #4 | (((((((community health services[MeSH Terms]) OR (community health nursing[MeSH Terms])) OR ("community health care"[Text Word])) OR ("community health centers"[Text Word])) OR (home[Text Word])) OR (kindergarten[Text Word])) OR (environment[Text Word])) OR (day care[Text Word]) | 1,481,733 |
| #3 | ((((((problem behavior[MeSH Terms]) OR ("behavioral problem*"[Text Word])) OR ("emotional problem*"[Text Word])) OR ("problem*, behavioral"[Text Word])) OR ("emotional and behavioral problems"[Text Word])) OR ("behavioural problem*"[Text Word])) OR ("emotional and behavioural problems"[Text Word]) | 22,339 |
| #2 | ((((((((risk factors[MeSH Terms]) OR (risk[MeSH Terms])) OR (protective factors[MeSH Terms])) OR (risk assessment[MeSH Terms])) OR (risk*[Text Word])) OR (protective*[Text Word])) OR (predict*[Text Word])) OR (determinant*[Text Word])) OR (factor*[Text Word]) | 10,920,422 |
| #1 | ((((child, preschool[MeSH Terms]) OR ("preschool child"[Text Word])) OR ("preschool children"[Text Word])) OR (Preschooler*[Text Word])) OR ("early childhood"[Text Word]) | 1,067,593 |
