# Supplementary material for: Mapping factors associated with emotional and behavioral problems among preschool children: A scoping review protocol
Source: PLoS One. 2026 Jul 15;21(7):e0353520. doi: 10.1371/journal.pone.0353520 (PMC13372158; doi:10.1371/journal.pone.0353520)
Supplement: S3 File — (DOCX) [file pone.0353520.s004.docx]

### S4 File. Data extraction form

| **No** | **Author (s) and Year** | **Study Design** | **Participant** | **Setting** | **Measurement Instruments** | **Domain** | **Key Findings** |
| --- | --- | --- | --- | --- | --- | --- | --- |
|  |  |  |  |  |  |  |  |
|  |  |  |  |  |  |  |  |
