# Supplementary material for: Mapping factors associated with emotional and behavioral problems among preschool children: A scoping review protocol
Source: PLoS One. 2026 Jul 15;21(7):e0353520. doi: 10.1371/journal.pone.0353520 (PMC13372158; doi:10.1371/journal.pone.0353520)
Supplement: Attachment — Submitted filename: Response_to_Reviewers.docx [file pone.0353520.s006.docx]

**Response to Editor and Reviewers**

Manuscript Title	: Mapping factors associated with emotional and behavioral problems among preschool children: a scoping review protocol

Manuscript ID	: PONE-D-25-68682

Date	: April 30, 2026

Dear Editor and Reviewers of PLOS ONE,

We would like to sincerely thank the Academic Editor and the Reviewers for their careful evaluation and constructive feedback on our manuscript. We greatly appreciate the insightful and detailed comments, which have helped us substantially improve the clarity, rigor, and methodological transparency of our work. In response, we have carefully revised the manuscript, particularly strengthening the Introduction and Methods sections as recommended. All revisions have been clearly indicated in the tracked version. Below, we provide a detailed, point-by-point response to each comment.

1) **Responses to the Academic Editor**

Comment #1:

Two expert reviewers have provided reports. As you will see from their feedback presented below, it is necessary to clarify the introduction and method sections.

Response:

We are grateful for this important guidance. In response, we have substantially revised both the Introduction and Methods sections to improve clarity, strengthen the rationale, and enhance methodological transparency. Specifically, the Introduction has been rewritten to better articulate the research gap, theoretical framework, and the study's novelty. The Methods section has been revised to ensure consistency with JBI scoping review methodology and PRISMA-ScR reporting guidelines, including detailed procedures for study selection, data extraction, and data synthesis.

Revision made:

a. Introduction section revised to strengthen clarity, gap identification, theoretical justification, and novelty (Lines 54-140).
b. Methods section expanded for transparency and reproducibility (Lines 159-280).

Comment #2:

Please include captions for your Supporting Information files at the end of your manuscript, and update any in-text citations to match accordingly.

Response:

Thank you for this important instruction. The Supporting Information section has been reviewed and revised to ensure full compliance with the journal’s requirements. Captions have been standardized and presented at the end of the manuscript using the required format, file naming has been aligned with journal conventions, and in-text citations have been checked and updated where necessary to ensure consistency with the corresponding Supporting Information labels.

Revision made:

Supporting Information captions have been standardized, file names have been revised according to journal conventions, and in-text citations have been corrected to ensure full consistency with the corresponding labels (Lines 310-319).

2) **Responses to Reviewer 1**

Comment #1:

The submission presents a clear and timely rationale for conducting a scoping review on emotional and behavioral problems (EBPs) in preschool children, effectively situating the topic within a public health framework. The purpose of the review is well articulated, and the use of established methodological guidance (Peters, JBI, and PRISMA-ScR) strengthens the rigor and transparency of the proposed approach. The inclusion of multiple databases, diverse study designs, and no language restrictions demonstrates methodological breadth and an intention to capture the interdisciplinary nature of the literature. The planned use of independent reviewers and standardized data charting further enhances the credibility and reproducibility of the review. Overall, the protocol reflects strong alignment with best practices for scoping reviews and clearly communicates its contribution to organizing a fragmented evidence base.

Response:

We sincerely thank the reviewer for the positive and constructive evaluation of our manuscript. We appreciate the recognition of the methodological rigor and relevance of our work.

Revision made:

No revision was required as this comment is supportive.

Comment #2:

While the four-factor domains (genetic, biological, psychological, and environmental) provide a useful organizing framework, a brief justification for this categorization-grounded in theory or prior models-would enhance conceptual clarity.

Response:

We are grateful for this valuable suggestion. The manuscript has been revised to provide a clear theoretical justification for the four-domain categorization, grounded in the biopsychosocial model and further supported by a developmental perspective. This revision strengthens the conceptual coherence of the study and improves the analytical structure used for mapping evidence.

The sentences added in the manuscript are as follows:

“The inclusion of genetic, biological, psychological, and environmental domains reflects an effort to operationalize the biopsychosocial framework into analytically distinct yet interrelated categories for evidence mapping. The biological domain is further differentiated within this framework to distinguish genetic factors from broader biological influences, enhancing conceptual precision and analytical clarity. This approach is further supported by a developmental perspective, including Bronfenbrenner’s bioecological model, which emphasizes the interaction between individual characteristics and environmental contexts across time.”

Revision made:

Theoretical justification has been added to the Introduction (Line 117-124).

Comment #3:

The protocol could also benefit from more explicit definitions of “emotional and behavioral problems” and how variability in measurement across studies will be addressed during synthesis.

Response:

We are grateful for this important suggestion. The manuscript has been revised to provide clearer definitions of emotional and behavioral problems (EBPs), explicitly distinguishing internalizing and externalizing domains, and to clarify how variability in measurement will be addressed.

The sentences added in the manuscript are as follows:

“EBPs in preschool children refer to persistent difficulties in age-appropriate and adaptive regulation of emotions and behaviors, encompassing both internalizing and externalizing domains. Internalizing problems include symptoms such as anxiety, depression, emotional withdrawal, and excessive fear, whereas externalizing problems include hyperactivity, inattention, aggression, oppositional behavior, and difficulties in social interaction, such as sharing and cooperating with peers.”

“… Studies will be eligible regardless of baseline status, provided that emotional and/or behavioral problems are examined as outcomes or phenomena of interest using standardized screening or assessment tools such as the Child Behavior Checklist (CBCL), Strengths and Difficulties Questionnaire (SDQ), Pediatric Symptom Checklist-17 (PSC-17), or comparable instruments.”

Revision made:

a. Expanded definition of EBPs in the Introduction (Lines 62-67)
b. Clarified synthesis approach in Methods (Lines 174-178)

Comment #4:

Finally, although the descriptive synthesis is appropriate for the scoping review, specifying how gaps and patterns will be analytically identified (e.g., frequency mapping or thematic grouping) would further strengthen the methodological transparency and usefulness of the findings for researchers and practitioners.

Response:

We appreciate this constructive suggestion. The manuscript has been revised to explicitly describe the analytical strategies used to identify patterns and gaps.

The sentences added in the manuscript are as follows:

“To enhance analytical rigor, frequency mapping will be used to quantify the distribution of evidence across domains, while thematic grouping will be applied to categorize the associated factors. A thematic synthesis approach will be used for qualitative studies, in which data will be inductively coded to identify recurring concepts, grouped into themes, and subsequently mapped onto the four conceptual domains.”

Revision made:

The Data Analysis and Presentation section expanded (Lines 266-271)

3) **Response to Reviewer 2**

Comment #1:

I appreciate the opportunity to review the protocol titled “Mapping Factors Associated with Emotional and Behavioral Problems Among Preschool Children: A Scoping Review Protocol.” The authors address an important topic with clear relevance to early childhood and special education, particularly for preschool children experiencing emotional and behavioral difficulties. While the protocol follows PRISMA-ScR guidelines and outlines the planned procedures for the scoping review, several concerns should be addressed prior to publication. First, the introduction lacks clarity, making the significance of the review somewhat unclear. Although the authors describe evidence linking early emotional and behavioral problems to later academic outcomes and well-being, it is not clear whether prior review studies have examined similar topics. If such reviews exist, what have they found? More importantly, what specific gaps in the existing literature does this review aim to address? Clarifying the novelty and contribution of the proposed review would strengthen the rationale.”

Response:

We thank the reviewer for this insightful and constructive comment, which has significantly strengthened the clarity and rationale of our manuscript. In response, we have substantially revised the Introduction to improve clarity, explicitly identify the existence of prior review studies, summarize their main findings and limitations, clearly articulate the research gap, and strengthen the novelty and study aim of the present scoping review.

Specifically, we now clarify that previous review studies have primarily focused on specific populations, conditions, or limited domains such as psychological or parenting-related factors and have generally been conducted as systematic reviews or meta-analyses with a relatively narrow scope. As a result, these reviews do not provide an integrative synthesis across multiple domains relevant to preschool children.

We further emphasize that existing literature is fragmented due to inconsistencies in factor categorization and methodological variability, limiting a comprehensive understanding of EBPs within the preschool developmental context.

To address this gap, we explicitly state the novelty of our approach in adopting a biopsychosocial and developmental framework to systematically map genetic, biological, psychological, and environmental factors within a unified analytical structure, using a scoping review methodology.

The sentences added in the manuscript are as follows:

“A growing body of research has examined factors associated with EBPs across different populations and settings. … However, much of the existing literature focuses on isolated factors, specific domains, or older age groups, thereby limiting a comprehensive understanding of EBPs within the unique developmental context of preschool children.”

“Previous review studies have examined EBPs within specific contexts or populations, such as disordered eating behavior, foster care settings, or parental stress, often focusing on limited sets of factors or age groups. Most of these studies have been conducted as systematic reviews or meta-analyses with a relatively narrow scope, rather than as comprehensive scoping reviews designed to map the full breadth of evidence across multiple domains ... Inconsistencies in the categorization of influencing factors and variability in methodological approaches have contributed to fragmentation in the existing evidence base.”

“Therefore, this scoping review aims to map and synthesize the existing literature on factors associated with EBPs in preschool children across genetic, biological, psychological, and environmental domains. This further seeks to describe how these factors have been examined and reported across studies, without implying causal relationships. This review provides an integrated and theoretically grounded overview of these factors to enhance conceptual clarity, identify research gaps, and inform future research, policy, and early intervention strategies.”

Revision made:

a. Added prior review and their limitations in the Introduction (Lines 91-99)
b. Added findings of prior reviews and research gap in the Introduction (Line 100-111)
c. Added novelty and study aim in the Introduction (Line 131-137)

Comment #2:

Second, the authors aim to address research questions focused on four components-genetic, biological, psychological, and environmental factors. However, the rationale for selecting these four domains is not sufficiently explained. Is there a theoretical framework guiding this categorization? Providing a clear conceptual or theoretical foundation would enhance the coherence of the review.

Response:

We appreciate this valuable suggestion. The manuscript has been revised to provide a clear theoretical foundation for the four-domain categorization. This framework is grounded in the biopsychosocial model and supported by Bronfenbrenner’s bioecological theory, emphasizing interactions between biological, psychological, and environmental systems across early development. This strengthens conceptual coherence and analytical rigor.

The sentences added in the manuscript are as follows:

“The inclusion of genetic, biological, psychological, and environmental domains reflects an effort to operationalize the biopsychosocial framework into analytically distinct yet interrelated categories for evidence mapping. The biological domain is further differentiated within this framework to distinguish genetic factors from broader biological influences, enhancing conceptual precision and analytical clarity. This approach is further supported by a developmental perspective, including Bronfenbrenner’s bioecological model, which emphasizes the interaction between individual characteristics and environmental contexts across time.”

Revision made:

Added theoretical framework in the Introduction (Lines 117-124)

Comment #3:

Third, regarding methodology, the protocol states that two reviewers will independently screen and extract data. Additional details are needed about the training of the second reviewer. What proportion of studies will be double-screened? What measure of inter-rater agreement will be calculated, and based on which formula? The authors also indicate that language will not be used as a screening criterion. If non-English studies are included, how will translation be conducted to ensure accuracy and consistency? Furthermore, since qualitative studies are included, the authors should clarify how qualitative evidence will be synthesized to address the research questions, particularly in identifying and mapping “factors” associated with emotional and behavioral problems.”

Response:

Thank you for this comprehensive and highly constructive comment. This feedback has helped us strengthen the manuscript's methodological transparency. In response, we have expanded the Methods section to address each methodological point raised: review training, the proportion of double-screening, inter-rater agreement and its calculation, handling of non-English studies, and qualitative evidence synthesis. First, reviewer training is conducted through a calibration exercise using approximately 5-10% of retrieved records, ensuring a shared understanding of the screening criteria. Second, study selection will be conducted by two reviewers who independently screen records at both title/abstract and full-text stages, as described in the Study selection section. Third, inter-rater agreements will be assessed using percentage agreement (target ≥90%) and Cohen’s kappa coefficient 0.61-0.80 as complementary measures of agreement beyond chance, interpreted using established frameworks (e.g., Landis and Koch). Fourth, no language restrictions are applied. Non-English studies will be translated using a reliable tool and verified where possible, with key sections cross-checked for accuracy and consistency. Fifth, qualitative data will be synthesized using a thematic approach, involving inductive coding, development of themes, and mapping onto the four predefined domains.

The sentences added in the manuscript are as follows:

“Two reviewers (WR and BM) will independently screen the records following a pilot calibration exercise to ensure consistency with the predefined inclusion criteria. Before the screening process, a calibration exercise will be conducted using a random sample of approximately 5-10% of the retrieved records, consistent with the methodological guidance of the scoping review. Both reviewers will independently screen these records, followed by a discussion to resolve discrepancies and refine the inclusion and exclusion criteria. If a high level of agreement is not achieved (e.g., below 90%), further calibration will be performed before proceeding [53]. This process serves as training and ensures a shared understanding of screening procedures.”

“Inter-rater agreement will be assessed using percentage agreement (target ≥90%) as the primary indicator of consistency during calibration [53]. Cohen’s kappa coefficient will also be calculated as a complementary measure to account for agreement beyond chance. Interpretation of the kappa value will follow Landis and Koch, where values of 0.61-0.80 indicate substantial agreement and values above 0.80 indicate almost perfect agreement [54].”

“The handling of non-English studies will be integrated within the data extraction procedures. No language restriction will be applied. For studies published in languages other than English, the initial translation will be conducted using a reliable translation tool (e.g., Google Translate), followed by verification by a reviewer with proficiency in the respective language, where possible. Key sections (e.g., methods and results) will be cross-checked when necessary to ensure data extraction accuracy and consistency.”

“A thematic synthesis approach will be used for qualitative studies, in which data will be inductively coded to identify recurring concepts, grouped into themes, and subsequently mapped onto the four conceptual domains.”

Revision made:

a. Added reviewer training and calibration procedures (Lines 224-233) and independent screening at both title/abstract and full-text (Lines 229-232)
b. Added inter-rater agreement (Line 234-239)
c. Added translation procedures for non-English studies (Lines 200-205)
d. Expanded qualitative synthesis approach using thematic group (Line 268-271)

Comment #4:

Overall, this protocol has the potential to make a meaningful contribution to the field. I appreciate the authors’ efforts in addressing this important topic and wish them success in their future work.

Response:

We sincerely thank the reviewer for this encouraging and supportive comment.

Revision made:

No revision was required as this comment is supportive.

We hope that the revisions satisfactorily address all comments, and we would be pleased to provide any further clarification if needed.

Thank you for your consideration.

Sincerely,
**Esme Anggeriyane**

on behalf of all authors

^1^Doctoral Study Program, Faculty of Medicine, Universitas Padjadjaran, Bandung, West Java, Indonesia
Email: [esme24001@mail.unpad.ac.id](mailto:esme24001@mail.unpad.ac.id)
