# Supplementary material for: Mapping factors associated with emotional and behavioral problems among preschool children: A scoping review protocol
Source: PLoS One. 2026 Jul 15;21(7):e0353520. doi: 10.1371/journal.pone.0353520 (PMC13372158; doi:10.1371/journal.pone.0353520)
Supplement: Peer review history

# Peer review: Decision Letter 0

**DOI:** 10.1371/journal.pone.0353520.r001

**Authors:** Catarina Canário

21 Apr 2026

PONE-D-25-68682

Mapping factors associated with emotional and behavioral problems among preschool children: a scoping review protocol

PLOS One

Dear Dr. Anggeriyane,

Thank you for submitting your manuscript to PLOS ONE. After careful consideration, we feel that it has merit but does not fully meet PLOS ONE’s publication criteria as it currently stands. Therefore, we invite you to submit a revised version of the manuscript that addresses the points raised during the review process.

Two expert reviewers have provided reports. As you will see from their feedback resented below, it is necessary to clarify the introduction and method sections.

Reviewer#1:

The submission presents a clear and timely rationale for conducting a scoping review on emotional and behavioral problems (EBPs) in preschool children, effectively situating the topic within a public health framework. The purpose of the review is well articulated, and the use of established methodological guidance (Peters, JBI, and PRISMA-ScR) strengthens the rigor and transparency of the proposed approach. The inclusion of multiple databases, diverse study designs, and no language restrictions demonstrates methodological breadth and an intention to capture the interdisciplinary nature of the literature. The planned use of independent reviewers and standardized data charting further enhances the credibility and reproducibility of the review. Overall, the protocol reflects strong alignment with best practices for scoping reviews and clearly communicates its contribution to organizing a fragmented evidence base.

While the four-factor domains (genetic, biological, psychological, and environmental) provide a useful organizing framework, a brief justification for this categorization—grounded in theory or prior models—would enhance conceptual clarity. The protocol could also benefit from more explicit definitions of “emotional and behavioral problems” and how variability in measurement across studies will be addressed during synthesis. Finally, although the descriptive synthesis is appropriate for a scoping review, specifying how gaps and patterns will be analytically identified (e.g., frequency mapping or thematic grouping) would further strengthen the methodological transparency and usefulness of the findings for researchers and practitioners.

Reviewer#2:

I appreciate the opportunity to review the protocol titled “Mapping Factors Associated with Emotional and Behavioral Problems Among Preschool Children: A Scoping Review Protocol.” The authors address an important topic with clear relevance to early childhood and special education, particularly for preschool children experiencing emotional and behavioral difficulties. While the protocol follows PRISMA-ScR guidelines and outlines the planned procedures for the scoping review, several concerns should be addressed prior to publication.

First, the introduction lacks clarity, making the significance of the review somewhat unclear. Although the authors describe evidence linking early emotional and behavioral problems to later academic outcomes and well-being, it is not clear whether prior review studies have examined similar topics. If such reviews exist, what have they found? More importantly, what specific gaps in the existing literature does this review aim to address? Clarifying the novelty and contribution of the proposed review would strengthen the rationale.

Second, the authors aim to address research questions focused on four components—genetic, biological, psychological, and environmental factors. However, the rationale for selecting these four domains is not sufficiently explained. Is there a theoretical framework guiding this categorization? Providing a clear conceptual or theoretical foundation would enhance the coherence of the review.

Third, regarding methodology, the protocol states that two reviewers will independently screen and extract data. Additional detail is needed about the training of the second reviewer. What proportion of studies will be double-screened? What measure of inter-rater agreement will be calculated, and based on which formula? The authors also indicate that language will not be used as a screening criterion. If non-English studies are included, how will translation be conducted to ensure accuracy and consistency? Furthermore, since qualitative studies are included, the authors should clarify how qualitative evidence will be synthesized to address the research questions, particularly in identifying and mapping “factors” associated with emotional and behavioral problems.

Overall, this protocol has the potential to make a meaningful contribution to the field. I appreciate the authors’ efforts in addressing this important topic and wish them success in their future work.

Please submit your revised manuscript by Jun 05 2026 11:59PM. If you will need more time than this to complete your revisions, please reply to this message or contact the journal office at plosone@plos.org. When you're ready to submit your revision, log on to https://www.editorialmanager.com/pone/ and select the 'Submissions Needing Revision' folder to locate your manuscript file.

Please include the following items when submitting your revised manuscript:

- A letter that responds to each point raised by the academic editor and reviewer(s). You should upload this letter as a separate file labeled 'Response to Reviewers'.
- A marked-up copy of your manuscript that highlights changes made to the original version. You should upload this as a separate file labeled 'Revised Manuscript with Track Changes'.
- An unmarked version of your revised paper without tracked changes. You should upload this as a separate file labeled 'Manuscript'.

If you would like to make changes to your financial disclosure, please include your updated statement in your cover letter. Guidelines for resubmitting your figure files are available below the reviewer comments at the end of this letter.

If applicable, we recommend that you deposit your laboratory protocols in protocols.io to enhance the reproducibility of your results. Protocols.io assigns your protocol its own identifier (DOI) so that it can be cited independently in the future. For instructions see: https://journals.plos.org/plosone/s/submission-guidelines#loc-laboratory-protocols. Additionally, PLOS ONE offers an option for publishing peer-reviewed Lab Protocol articles, which describe protocols hosted on protocols.io. Read more information on sharing protocols at https://plos.org/protocols?utm_medium=editorial-email&utm_source=authorletters&utm_campaign=protocols.

As the corresponding author, your ORCID iD is verified in the submission system and will appear in the published article. PLOS supports the use of ORCID, and we encourage all coauthors to register for an ORCID iD and use it as well. Please encourage your coauthors to verify their ORCID iD within the submission system before final acceptance, as unverified ORCID iDs will not appear in the published article. *Only*  the individual author can complete the verification step; PLOS staff *cannot*  verify ORCID iDs on behalf of authors.

We look forward to receiving your revised manuscript.

Kind regards,

Catarina Canário

Academic Editor

PLOS One

Journal requirements:

When submitting your revision, we need you to address these additional requirements.

1. Please ensure that your manuscript meets PLOS ONE's style requirements, including those for file naming. The PLOS ONE style templates can be found at

https://journals.plos.org/plosone/s/file?id=wjVg/PLOSOne_formatting_sample_main_body.pdf and

https://journals.plos.org/plosone/s/file?id=ba62/PLOSOne_formatting_sample_title_authors_affiliations.pdf

2. Please ensure that you include a title page within your main document. You should list all authors and all affiliations as per our author instructions and clearly indicate the corresponding author.

3. Please include captions for your Supporting Information files at the end of your manuscript, and update any in-text citations to match accordingly. Please see our Supporting Information guidelines for more information: http://journals.plos.org/plosone/s/supporting-information.

If the reviewer comments include a recommendation to cite specific previously published works, please review and evaluate these publications to determine whether they are relevant and should be cited. There is no requirement to cite these works unless the editor has indicated otherwise.

Additional Editor Comments:

Dear Dr. Anggeriyane,

Thank you very much for submitting your manuscript “Mapping factors associated with emotional and behavioral problems among preschool children: a scoping review protocol" to PLOS One. Two expert reviewers have provided reports. As you will see from their feedback resented below, it is necessary to clarify the introduction and method sections.

Based on their feedback, I invite you to revise and resubmit your manuscript by May 31th, 2026. Please include a point-by-point reply, explaining how you incorporated the feedback into the revised version of your manuscript.

We are looking forward to receiving your revised manuscript.

Best wishes,

Reviewer#1:

The submission presents a clear and timely rationale for conducting a scoping review on emotional and behavioral problems (EBPs) in preschool children, effectively situating the topic within a public health framework. The purpose of the review is well articulated, and the use of established methodological guidance (Peters, JBI, and PRISMA-ScR) strengthens the rigor and transparency of the proposed approach. The inclusion of multiple databases, diverse study designs, and no language restrictions demonstrates methodological breadth and an intention to capture the interdisciplinary nature of the literature. The planned use of independent reviewers and standardized data charting further enhances the credibility and reproducibility of the review. Overall, the protocol reflects strong alignment with best practices for scoping reviews and clearly communicates its contribution to organizing a fragmented evidence base.

While the four-factor domains (genetic, biological, psychological, and environmental) provide a useful organizing framework, a brief justification for this categorization—grounded in theory or prior models—would enhance conceptual clarity. The protocol could also benefit from more explicit definitions of “emotional and behavioral problems” and how variability in measurement across studies will be addressed during synthesis. Finally, although the descriptive synthesis is appropriate for a scoping review, specifying how gaps and patterns will be analytically identified (e.g., frequency mapping or thematic grouping) would further strengthen the methodological transparency and usefulness of the findings for researchers and practitioners.

Reviewer#2:

I appreciate the opportunity to review the protocol titled “Mapping Factors Associated with Emotional and Behavioral Problems Among Preschool Children: A Scoping Review Protocol.” The authors address an important topic with clear relevance to early childhood and special education, particularly for preschool children experiencing emotional and behavioral difficulties. While the protocol follows PRISMA-ScR guidelines and outlines the planned procedures for the scoping review, several concerns should be addressed prior to publication.

First, the introduction lacks clarity, making the significance of the review somewhat unclear. Although the authors describe evidence linking early emotional and behavioral problems to later academic outcomes and well-being, it is not clear whether prior review studies have examined similar topics. If such reviews exist, what have they found? More importantly, what specific gaps in the existing literature does this review aim to address? Clarifying the novelty and contribution of the proposed review would strengthen the rationale.

Second, the authors aim to address research questions focused on four components—genetic, biological, psychological, and environmental factors. However, the rationale for selecting these four domains is not sufficiently explained. Is there a theoretical framework guiding this categorization? Providing a clear conceptual or theoretical foundation would enhance the coherence of the review.

Third, regarding methodology, the protocol states that two reviewers will independently screen and extract data. Additional detail is needed about the training of the second reviewer. What proportion of studies will be double-screened? What measure of inter-rater agreement will be calculated, and based on which formula? The authors also indicate that language will not be used as a screening criterion. If non-English studies are included, how will translation be conducted to ensure accuracy and consistency? Furthermore, since qualitative studies are included, the authors should clarify how qualitative evidence will be synthesized to address the research questions, particularly in identifying and mapping “factors” associated with emotional and behavioral problems.

Overall, this protocol has the potential to make a meaningful contribution to the field. I appreciate the authors’ efforts in addressing this important topic and wish them success in their future work.

[Note: HTML markup is below. Please do not edit.]

Reviewers' comments:

Reviewer's Responses to Questions

**Comments to the Author**

1. Does the manuscript provide a valid rationale for the proposed study, with clearly identified and justified research questions?

The research question outlined is expected to address a valid academic problem or topic and contribute to the base of knowledge in the field.

Reviewer #1: Yes

Reviewer #2: Yes

**********

2. Is the protocol technically sound and planned in a manner that will lead to a meaningful outcome and allow testing the stated hypotheses?

The manuscript should describe the methods in sufficient detail to prevent undisclosed flexibility in the experimental procedure or analysis pipeline, including sufficient outcome-neutral conditions (e.g. necessary controls, absence of floor or ceiling effects) to test the proposed hypotheses and a statistical power analysis where applicable. As there may be aspects of the methodology and analysis which can only be refined once the work is undertaken, authors should outline potential assumptions and explicitly describe what aspects of the proposed analyses, if any, are exploratory.

Reviewer #1: Yes

Reviewer #2: Partly

**********

3. Is the methodology feasible and described in sufficient detail to allow the work to be replicable?

Descriptions of methods and materials in the protocol should be reported in sufficient detail for another researcher to reproduce all experiments and analyses. The protocol should describe the appropriate controls, sample size calculations, and replication needed to ensure that the data are robust and reproducible.

Reviewer #1: Yes

Reviewer #2: No

**********

4. Have the authors described where all data underlying the findings will be made available when the study is complete?

The PLOS Data policy requires authors to make all data underlying the findings described in their manuscript fully available without restriction, with rare exception, at the time of publication. The data should be provided as part of the manuscript or its supporting information, or deposited to a public repository. For example, in addition to summary statistics, the data points behind means, medians and variance measures should be available. If there are restrictions on publicly sharing data—e.g. participant privacy or use of data from a third party—those must be specified.

Reviewer #1: Yes

Reviewer #2: Yes

**********

5. Is the manuscript presented in an intelligible fashion and written in standard English?

PLOS ONE does not copyedit accepted manuscripts, so the language in submitted articles must be clear, correct, and unambiguous. Any typographical or grammatical errors should be corrected at revision, so please note any specific errors here.

Reviewer #1: Yes

Reviewer #2: Yes

**********

6. Review Comments to the Author

Please use the space provided to explain your answers to the questions above and, if applicable, provide comments about issues authors must address before this protocol can be accepted for publication. You may also include additional comments for the author, including concerns about research or publication ethics.

You may also provide optional suggestions and comments to authors that they might find helpful in planning their study.

(Please upload your review as an attachment if it exceeds 20,000 characters)

Reviewer #1: The submission presents a clear and timely rationale for conducting a scoping review on emotional and behavioral problems (EBPs) in preschool children, effectively situating the topic within a public health framework. The purpose of the review is well articulated, and the use of established methodological guidance (Peters, JBI, and PRISMA-ScR) strengthens the rigor and transparency of the proposed approach. The inclusion of multiple databases, diverse study designs, and no language restrictions demonstrates methodological breadth and an intention to capture the interdisciplinary nature of the literature. The planned use of independent reviewers and standardized data charting further enhances the credibility and reproducibility of the review. Overall, the protocol reflects strong alignment with best practices for scoping reviews and clearly communicates its contribution to organizing a fragmented evidence base.

While the four-factor domains (genetic, biological, psychological, and environmental) provide a useful organizing framework, a brief justification for this categorization—grounded in theory or prior models—would enhance conceptual clarity. The protocol could also benefit from more explicit definitions of “emotional and behavioral problems” and how variability in measurement across studies will be addressed during synthesis. Finally, although the descriptive synthesis is appropriate for a scoping review, specifying how gaps and patterns will be analytically identified (e.g., frequency mapping or thematic grouping) would further strengthen the methodological transparency and usefulness of the findings for researchers and practitioners.

Reviewer #2: I appreciate the opportunity to review the protocol titled “Mapping Factors Associated with Emotional and Behavioral Problems Among Preschool Children: A Scoping Review Protocol.” The authors address an important topic with clear relevance to early childhood and special education, particularly for preschool children experiencing emotional and behavioral difficulties. While the protocol follows PRISMA-ScR guidelines and outlines the planned procedures for the scoping review, several concerns should be addressed prior to publication.

First, the introduction lacks clarity, making the significance of the review somewhat unclear. Although the authors describe evidence linking early emotional and behavioral problems to later academic outcomes and well-being, it is not clear whether prior review studies have examined similar topics. If such reviews exist, what have they found? More importantly, what specific gaps in the existing literature does this review aim to address? Clarifying the novelty and contribution of the proposed review would strengthen the rationale.

Second, the authors aim to address research questions focused on four components—genetic, biological, psychological, and environmental factors. However, the rationale for selecting these four domains is not sufficiently explained. Is there a theoretical framework guiding this categorization? Providing a clear conceptual or theoretical foundation would enhance the coherence of the review.

Third, regarding methodology, the protocol states that two reviewers will independently screen and extract data. Additional detail is needed about the training of the second reviewer. What proportion of studies will be double-screened? What measure of inter-rater agreement will be calculated, and based on which formula? The authors also indicate that language will not be used as a screening criterion. If non-English studies are included, how will translation be conducted to ensure accuracy and consistency? Furthermore, since qualitative studies are included, the authors should clarify how qualitative evidence will be synthesized to address the research questions, particularly in identifying and mapping “factors” associated with emotional and behavioral problems.

Overall, this protocol has the potential to make a meaningful contribution to the field. I appreciate the authors’ efforts in addressing this important topic and wish them success in their future work.

**********

7. PLOS authors have the option to publish the peer review history of their article (what does this mean?). If published, this will include your full peer review and any attached files.

If you choose “no”, your identity will remain anonymous but your review may still be made public.

**Do you want your identity to be public for this peer review?**  For information about this choice, including consent withdrawal, please see our Privacy Policy.

Reviewer #1: No

Reviewer #2: No

**********

[NOTE: If reviewer comments were submitted as an attachment file, they will be attached to this email and accessible via the submission site. Please log into your account, locate the manuscript record, and check for the action link "View Attachments". If this link does not appear, there are no attachment files.]

To ensure your figures meet our technical requirements, please review our figure guidelines: https://journals.plos.org/plosone/s/figures

You may also use PLOS’s free figure tool, NAAS, to help you prepare publication quality figures: https://journals.plos.org/plosone/s/figures#loc-tools-for-figure-preparation.

NAAS will assess whether your figures meet our technical requirements by comparing each figure against our figure specifications.

# Author comment: Author response to Decision Letter 1

**DOI:** 10.1371/journal.pone.0353520.r002

30 Apr 2026

Dear Reviewers,

We sincerely thank you for your insightful and constructive comments. We have carefully addressed all specific points raised and revised the manuscript accordingly.

In response to your comments, we have:

1) Clarified the introduction by strengthening the study rationale, incorporating relevant previous review findings, and explicitly identifying the research gap;

2) Provided a clearer theoretical justification for the categorization of factors into genetic, biological, psychological, and environmental domains;

3) Clarified the definition of emotional and behavioral problems and how variability in measurement across studies will be addressed;

4) Expanded the Methods section to include detailed descriptions of reviewer training, screening procedures, inter-rater agreement, and handling of non-English studies; and

5) Specified the analytical approach for identifying patterns and gaps, including mapping and thematic grouping of findings, as well as the approach to qualitative evidence synthesis.

A detailed, point-by-point response to each comment is provided in the "Response to Reviewer" document, and all revisions have been clearly indicated in the tracked changes version of the manuscript.

We hope that these revisions have adequately addressed your concerns and improved the clarity and rigor of the manuscript.

Kind regards,

Esme Anggeriyane

Attachment Submitted filename: Response_to_Reviewers.docx

# Peer review: Decision Letter 1

**DOI:** 10.1371/journal.pone.0353520.r003

**Authors:** Catarina Canário

25 Jun 2026

Mapping factors associated with emotional and behavioral problems among preschool children: a scoping review protocol

PONE-D-25-68682R1

Dear Dr. Anggeriyane,

We’re pleased to inform you that your manuscript has been judged scientifically suitable for publication and will be formally accepted for publication once it meets all outstanding technical requirements.

Within one week, you’ll receive an e-mail detailing the required amendments. When these have been addressed, you’ll receive a formal acceptance letter and your manuscript will be scheduled for publication.

An invoice will be generated when your article is formally accepted. Please note, if your institution has a publishing partnership with PLOS and your article meets the relevant criteria, all or part of your publication costs will be covered. Please make sure your user information is up-to-date by logging into Editorial Manager at Editorial Manager® and clicking the ‘Update My Information' link at the top of the page. For questions related to billing, please contact billing support.

If your institution or institutions have a press office, please notify them about your upcoming paper to help maximize its impact. If they’ll be preparing press materials, please inform our press team as soon as possible -- no later than 48 hours after receiving the formal acceptance. Your manuscript will remain under strict press embargo until 2 pm Eastern Time on the date of publication. For more information, please contact onepress@plos.org.

Kind regards,

Catarina Canário

Academic Editor

PLOS One

Reviewers' comments:

Reviewer's Responses to Questions

**Comments to the Author**

1. Does the manuscript provide a valid rationale for the proposed study, with clearly identified and justified research questions?

The research question outlined is expected to address a valid academic problem or topic and contribute to the base of knowledge in the field.

Reviewer #1: Yes

**********

2. Is the protocol technically sound and planned in a manner that will lead to a meaningful outcome and allow testing the stated hypotheses?

The manuscript should describe the methods in sufficient detail to prevent undisclosed flexibility in the experimental procedure or analysis pipeline, including sufficient outcome-neutral conditions (e.g. necessary controls, absence of floor or ceiling effects) to test the proposed hypotheses and a statistical power analysis where applicable. As there may be aspects of the methodology and analysis which can only be refined once the work is undertaken, authors should outline potential assumptions and explicitly describe what aspects of the proposed analyses, if any, are exploratory.

Reviewer #1: Yes

**********

3. Is the methodology feasible and described in sufficient detail to allow the work to be replicable?

Descriptions of methods and materials in the protocol should be reported in sufficient detail for another researcher to reproduce all experiments and analyses. The protocol should describe the appropriate controls, sample size calculations, and replication needed to ensure that the data are robust and reproducible.

Reviewer #1: Yes

**********

4. Have the authors described where all data underlying the findings will be made available when the study is complete?

The PLOS Data policy requires authors to make all data underlying the findings described in their manuscript fully available without restriction, with rare exception, at the time of publication. The data should be provided as part of the manuscript or its supporting information, or deposited to a public repository. For example, in addition to summary statistics, the data points behind means, medians and variance measures should be available. If there are restrictions on publicly sharing data—e.g. participant privacy or use of data from a third party—those must be specified.

Reviewer #1: Yes

**********

5. Is the manuscript presented in an intelligible fashion and written in standard English?

PLOS ONE does not copyedit accepted manuscripts, so the language in submitted articles must be clear, correct, and unambiguous. Any typographical or grammatical errors should be corrected at revision, so please note any specific errors here.

Reviewer #1: Yes

**********

6. Review Comments to the Author

Please use the space provided to explain your answers to the questions above and, if applicable, provide comments about issues authors must address before this protocol can be accepted for publication. You may also include additional comments for the author, including concerns about research or publication ethics.

You may also provide optional suggestions and comments to authors that they might find helpful in planning their study.

(Please upload your review as an attachment if it exceeds 20,000 characters)

Reviewer #1: The authors have addressed the reviewer feedback thoroughly and appropriately in the revised manuscript. The revisions strengthen the clarity and methodological rigor of the proposed scoping review by providing a more coherent rationale, refining the description of the review procedures, and aligning the protocol with established Joanna Briggs Institute (JBI) and PRISMA-ScR guidelines. The manuscript now presents a clearer explanation of the search strategy, study selection process, data charting procedures, and planned synthesis methods, enhancing transparency and reproducibility. Furthermore, the organization of factors into genetic, biological, psychological, and environmental domains contributes to a more systematic framework for examining emotional and behavioral problems in preschool children. Overall, the revisions satisfactorily address the concerns raised during the initial review and improve the manuscript’s contribution to the literature on early childhood mental health.

**********

7. PLOS authors have the option to publish the peer review history of their article (what does this mean?). If published, this will include your full peer review and any attached files.

If you choose “no”, your identity will remain anonymous but your review may still be made public.

**Do you want your identity to be public for this peer review?**  For information about this choice, including consent withdrawal, please see our Privacy Policy.

Reviewer #1: No

**********

# Editor report: Acceptance letter

**DOI:** 10.1371/journal.pone.0353520.r004

**Authors:** Catarina Canário

PONE-D-25-68682R1

PLOS One

Dear Dr. Anggeriyane,

I'm pleased to inform you that your manuscript has been deemed suitable for publication in PLOS One. Congratulations! Your manuscript is now being handed over to our production team.

At this stage, our production department will prepare your paper for publication. This includes ensuring the following:

* All references, tables, and figures are properly cited

* All relevant supporting information is included in the manuscript submission,

* There are no issues that prevent the paper from being properly typeset

You will receive further instructions from the production team, including instructions on how to review your proof when it is ready. Please keep in mind that we are working through a large volume of accepted articles, so please give us a few days to review your paper and let you know the next and final steps.

Lastly, if your institution or institutions have a press office, please let them know about your upcoming paper now to help maximize its impact. If they'll be preparing press materials, please inform our press team within the next 48 hours. Your manuscript will remain under strict press embargo until 2 pm Eastern Time on the date of publication. For more information, please contact onepress@plos.org.

You will receive an invoice from PLOS for your publication fee after your manuscript has reached the completed accept phase. If you receive an email requesting payment before acceptance or for any other service, this may be a phishing scheme. Learn how to identify phishing emails and protect your accounts at https://explore.plos.org/phishing.

If we can help with anything else, please email us at customercare@plos.org.

Thank you for submitting your work to PLOS ONE and supporting open access.

Kind regards,

PLOS ONE Editorial Office Staff

on behalf of

Dr. Catarina Canário

Academic Editor

PLOS One
